# Determining k-Value with Regard to Freeze-Thaw Resistance of Concretes Containing GGBS

**DOI:** 10.3390/ma11122349

**Published:** 2018-11-22

**Authors:** Jerzy Wawrzeńczyk, Agnieszka Molendowska, Tomasz Juszczak

**Affiliations:** Faculty of Civil Engineering and Architecture, Kielce University of Technology, Al. Tysiąclecia Państwa Polskiego 7, 25-314 Kielce, Poland; agam@tu.kielce.pl (A.M.); tomasz.juszczak@dyckerhoff.com (T.J.)

**Keywords:** GGBS, k-value, freeze-thaw resistance, non-air-entrained concrete

## Abstract

The European concrete standard EN 206 introduces the k-value concept as one of the three methods allowing the use of granulated blast furnace slag in the design of the freeze-thaw-resistant concrete mix. It is assumed that the freeze-thaw durability of the concrete, whose composition (w/c ratio) has been corrected by adopting a certain k-value, is the same as the freeze-thaw resistance of the reference concrete made with the cement containing no addition (CEM I). This article presents the results of freeze-thaw resistance analysis (modified with the ASTM C666A standard Class XF3) of 24 series of concretes made with a binder containing varied amounts of slag, with a w/b ratio ranging from 0.25 to 0.55. The aim of the study was to estimate the k-value as a parameter defined by the w/b ratio and the slag content in the binder. In this approach, the k-value is determined by trial and error in such a way that the deformation of the concrete specimens containing the slag corresponds to the deformation of the reference concrete. As shown by the analysis, the k-value decreases with increasing slag content in the binder.

## 1. Introduction

According to the PN-EN 206 standard, the adoption of appropriate exposure classes and the correct design and manufacture of the concrete mix ensure adequate durability of concrete structures. It is important to use the right type of cement and w/b limiting value. The limit values regarding the composition and the properties of concrete defined in the standard are specified only for Portland cement CEM I, with other cement categories being ignored in this respect. 

In the case of hydrotechnical and bridge structures, where massive constructions are often found, it is technically and economically essential to use cements containing blast furnace slag addition. The basic technological requirement is to reduce the heat of cement hydration to prevent young concrete from damage as a result of thermal and shrinkage stresses. Such concrete must simultaneously meet a number of requirements regarding water permeability, resistance to chemical agents, and freeze-thaw resistance. Compressive strength is of secondary importance. 

The use of granulated blast furnace slag, assuming a sufficiently long period of moist curing and good compaction, results in decreased total porosity of hardened concrete. Analysis of the pore size distribution indicates a lower capillary pore volume and a general reduction in the average pore size. This decides about the improvement of the mechanical properties and increased durability of such concretes. 

Ground granulated blast furnace slag (GGBS) can be a constituent in pre-blended factory-produced cement or a separate component for concrete added directly to the concrete mix. The first solution is recommended, as the quality of the addition as well as its fineness are controlled, which guarantees the production of good quality cement. The PN-EN 206 standard specifies slag as a type II addition and introduces the concept of k-value for GGBS with the application rules the same as for fly ash and silica fume. A k-value of 0.6 is recommended when using the slag along with cements CEM I and CEM II/A. The maximum amount of slag used should meet the recommendation: GGBS/cement ≤1.0 by mass. If a greater amount of GGBS is used, the excess should not be taken into account for the calculation of the water/(cement + k × GGBS) ratio and the minimum cement content. The issue of adding granulated blast furnace slag directly to the concrete was addressed by Sanjuan et al. [1] and the following findings based on the literature review were reported:

- the k-value decreases as the amount of GGBS increases;
- the k-value increases with increasing fineness of GGBS (larger surface area increases GGBS hydraulic activity);
- k-values are higher in lower strength cements;
- GGBS concretes are more sensitive to poor curing conditions than concretes without addition;
- the expansion of GGBS concretes subjected to freeze-thaw cycles is much greater. Concrete expansion is greater even though an increased amount of GGBS can increase the 28-day compressive strength of concrete.

According to Sanjuan et al. [1], the adoption of a general k-value is both complicated and risky. The k-values recommended in the national standards and guidelines have to be defined with caution, assuming safe criteria. The values defined for one particular case provide no guarantee that the results will be repeatable in other applications. With the current knowledge of the problem, the k-value should be determined experimentally. Similar conclusions are contained in the work of Chromy et al. [2].

In the case of ordinary and high-performance concrete, the microstructure is the main determinant of mechanical properties, permeability, and durability [3]. The permeability of cementitious composites is closely related to the pore content, their structure, and the percentage of open pores.

The use of mineral additives is known to reduce permeability even when concrete is of a young age [4]. Very tight and impermeable concrete can be obtained by designing a concrete mix with mineral additions at a small w/c ratio of about 0.35 [5]. In the early period of hardening, the physical action of the filler consists in reducing porosity by filling the pores and decreasing their size.

The study by Stark and Ludwig [6] shows that the effects associated with slower hydration of slag cements can be compensated for by increasing the tightness of the paste, provided that a significant degree of hydration is achieved. It follows from the study that the GGBS-based paste at a hydration degree of α = 50% has a similar pore structure to that of Portland cement with α = 90%. When the degree of hydration exceeds 50%, the pastes containing GGBS have tighter microstructures than Ordinary Portland Cement (OPC) pastes. Achieving an adequate degree of hydration is primarily dependent on the quality of the slag, fineness of the cement, and curing conditions of the paste.

The key question is whether water-tight, low-permeability concrete requires air-entrainment to achieve good freeze-thaw resistance. The formation of cracks due to frost was explained quite a long time ago. As observed by Powers [7], upon freezing, water increases its volume and is pushed towards the air pores. At the same time, the water moves capillaries, where ice is formed, that is in the direction of cooler zones. On this basis, Powers and Helmuth [8] put forward a second hypothesis about osmotic pressure. With water freezing, the concentration of ions in the resultant solution increases, thereby creating osmotic pressure. Tensile stresses in the concrete grow at the rate of water movement—the rate of temperature drop and the distance to the nearest air pore. The logical conclusion is that the simplest way to prevent the formation of internal cracks due to freeze-thaw cycles is to properly air-entrain the concrete, while maintaining an adequate spacing between air pores. The idea is simple but its implementation in construction practice encounters a number of problems, as is the case with high-performance concrete (HPC), in which stable air entrainment and good air-pore distribution are difficult to achieve [5]. Pigeon et al. [9] point to the need for explaining the formation process of large air bubbles in the air-entrained concrete as air-entraining admixtures ensuring proper air volume often fail to provide adequate air-bubble spacing and, by extension, the required protection against freezing. 

A significant number of research papers are devoted to blast furnace cement. Deja [10] showed that the effectiveness of air-entraining admixture in a fresh concrete mix was much lower in the case of blast furnace cement concrete than in Portland cement concrete. To obtain the same air content, the addition of admixture was even twice as high as in the case of Portland cement concrete. The results reported by Giergiczny et al. [11] show that increasing the GGBS content in the cement results in higher compressive and flexural strength, reduced water absorption, and reduced depth of water penetration into the concrete. As for air-pore distribution in the hardened concrete, an increased content of slag causes a decrease in the total air volume and changes in the structure of these air pores resulting in a decrease in the content of micropores and an increase in the air-pore spacing of about 0.10 mm.

According to Peng et al. [12], air-entrainment is unnecessary when the w/b ratio is less than the critical value which determines achievement of impermeable concrete. Bilek et al. [13,14] show that the use of ternary binders may eliminate the need for air-entrainment. 

The PN-B-6265: 2004 standard, which is a supplement to the PN-EN 206-1 standard, allows the cements CEM I, CEM II/A-S, CEM II/B-S, CEM III/A, and CEM III/B (subject to some limitations for e.g., cement 32, 5R with up to 50% GGBS, as in ACI Committee 226 [15] requirements) to be applied to concretes of the required freeze-thaw resistance. 

The present work reviews the results of the research and so is a contribution to the discussion on the guidelines for the design of frost-resistant non-air-entrained concrete based on cements with different slag contents. The considerations focus on the XF3 exposure class, i.e., the concretes in structural elements, in which concrete can reach a high degree of saturation with water and can undergo freezing and thawing cycles. The study aimed at determining the k-value for concretes produced with different GGBS-containing binders within the context of its freeze-thaw resistance. 

## 2. Materials and Methods 

The test program included producing two series of non-air-entrained concretes:

- concretes made with cement CEM I, and three pre-blended cements: CEM II/A-S (13% GGBS), CEM II/B-S (28% GGBS), and CEM III/A (53% GGBS),
- concretes made with cement binders consisting of CEM I and GGBS addition in the range 0 to 60%.

The following materials were used in this study:

- cements: CEM I 42, 5R HSR NA, CEM II/A-S 42, 5N, CEM II/B-S 32, 5R, CEM III/A 32, 5N, and CEM I 42, 5R NA;
- GGBS;
- natural sand 0/2 mm;
- coarse basalt aggregate, fractions 4/8 and 8/16 mm;
- plasticizer.

Table 1 compiles the basic physical and chemical properties of the cement and GGBS. Aggregate grading was as follows: 32% sand, 33% 4–8 mm aggregate, 35% 8–16 mm aggregate.

Concrete mixes were prepared in a forced-mix laboratory mixer. First, the solid constituents were dosed and mixed for 60 s, then water and finally plasticizer were added, after which the mix was mixed for 90 s. The concrete testing on the homogeneous mixture included slump to PN-EN 12350-2:2011 and density to PN-EN 12390-7:2011.

The concrete mix was compacted on a vibrating table. The specimens were removed from the moulds after 24 h. The freeze-thaw resistance specimens were stored in water (at 20 ± 2 °C) until an age of 7 days and then in dry air (20 ± 2 °C, humidity 65 ± 5%) for 21 days. 

Table 2 shows the design of the experiment. The w/b ratios ranged from 0.25 to 0.55.

Two beams with dimensions 8 cm × 8 cm × 35 cm were used for freeze-thaw resistance testing according to the modified ASTM C666 method Procedure A. After 28 days of maturing, the specimens were saturated in water for 7 days. Then, the specimens were immersed in water in metal containers, in which they remained all the time during freezing and thawing at high degree of water saturation, corresponding to XF3 exposure class according to PN-EN 206. The tests included 300 freezing cycles at −18 ± 2 °C and thawing at +10 ± 2 °C, taking 3 cycles a day. Once a week, the specimens were removed from the freezing chamber and the degree of deterioration was determined based on observations of their appearance and mass and length measurements. The measurement of linear expansion was possible due to the attachment of metal plugs on the front surfaces of the beams during moulding. 

## 3. Results

**Figure 1:**
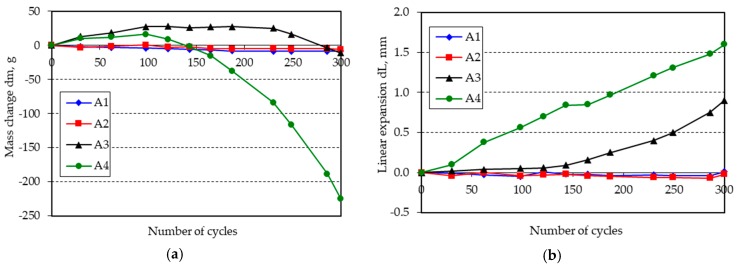
(**a**) Mass change; (**b**) linear expansion of specimens A1–A4 following freezing and thawing in water.

**Figure 2:**
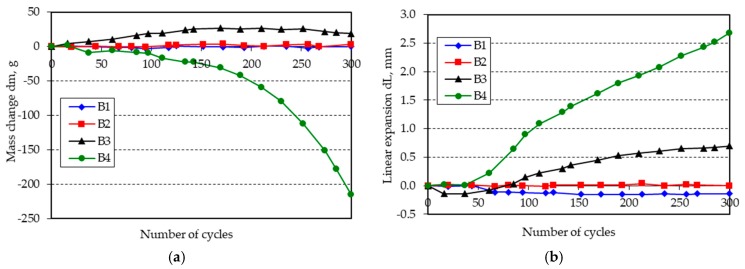
(**a**) Mass change; (**b**) linear expansion of specimens B1–B4 following freezing and thawing in water.

**Figure 3:**
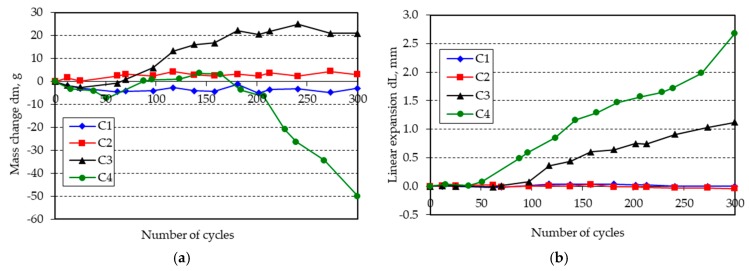
(**a**) Mass change; (**b**) linear expansion of specimens C1–C4 following freezing and thawing in water.

**Figure 4:**
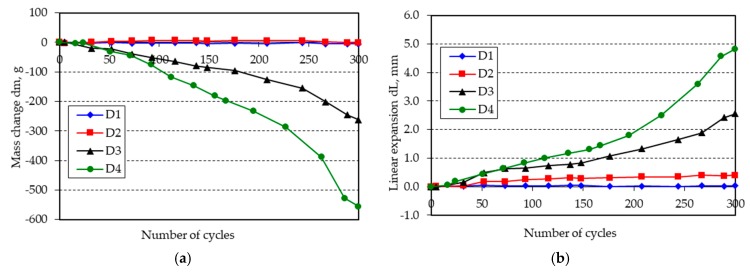
(**a**) Mass change; (**b**) linear expansion of specimens D1–D4 following freezing and thawing in water.

**Figure 5:**
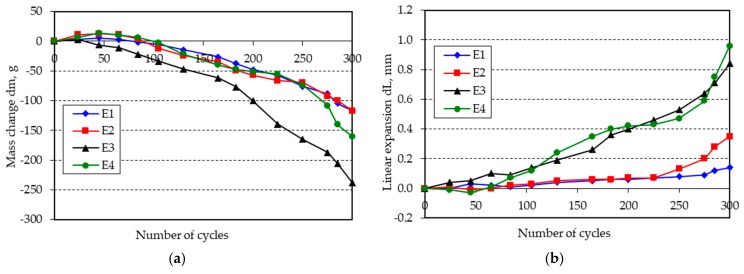
(**a**) Mass change; (**b**) linear expansion of specimens E1–E4 following freezing and thawing in water.

**Figure 6:**
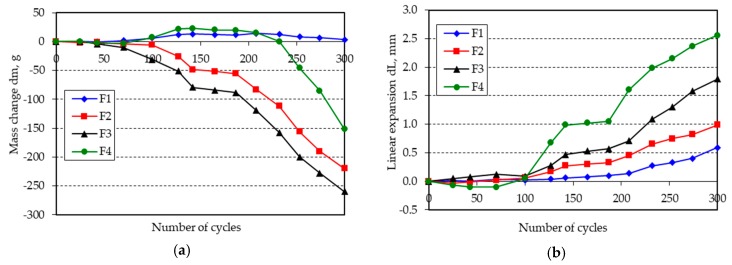
(**a**) Mass change; (**b**) linear expansion of specimens F1–F4 following freezing and thawing in water.

The changes in mass and length of the beam specimens after 300 freeze-thaw cycles in water are shown in Figure 1, Figure 2, Figure 3, Figure 4, Figure 5 and Figure 6.

## 4. Discussion

The freeze-thaw resistance assessment was carried out for non-air-entrained concretes made with cements of different GGBS content (A, B, C, D series) and for concretes made with CEM I cement and GGBS addition (E and F series). 

**Figure 7:**
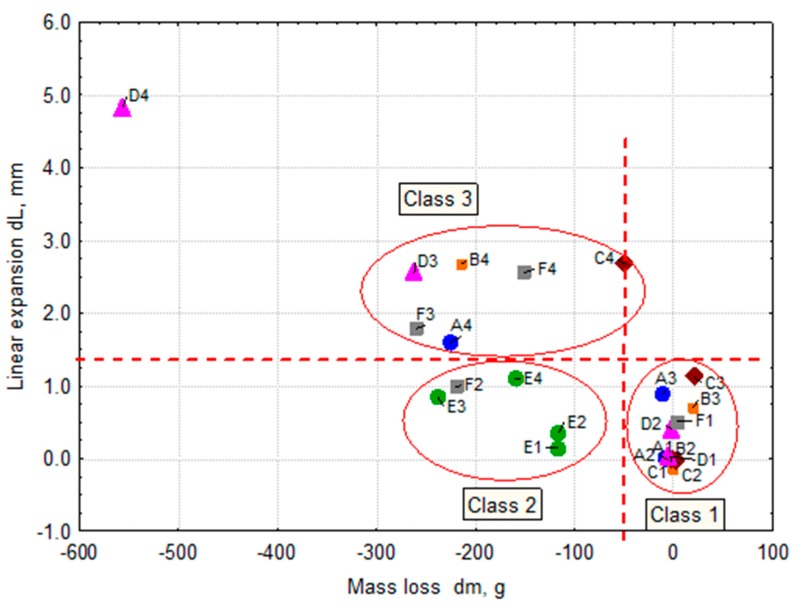
Relationship between the mass loss dm and linear expansion dL by frost resistance classes.

Figure 7 shows the results of the freeze-thaw resistance tests: mass changes dm and linear expansion dL for individual series of concrete specimens. Assuming the limiting values are dm = 50 g and dL = 1.3 mm, two lines divide the area of the diagram into subregions and three classes of freeze-thaw resistance can be distinguished:

- concretes with the best freeze-thaw resistance, for which small changes in mass dm and small linear expansion dL were recorded (class 1);
- a group of concretes with small linear expansion dL but with significant mass losses dm (scaling) (class 2);
- concretes with the lowest freeze-thaw resistance, where both mass loss dm and linear expansion dL were substantial (class 3).

Class 1, with the best freeze-thaw resistance, includes high-performance concrete (w/b ratio = 0.35) with a very tight microstructure. The mass change ranges from 0 to 21 g and the linear expansion dL is in the range 0 to 1.12 mm. Class 2 concretes are resistant to internal cracking but susceptible to scaling. Linear expansion dL is in the range 0.14 to 0.99 mm and the mass loss dm = 117–238 g. Class 3 includes non-freeze-thaw resistant concrete with the mass loss dm = 50–556 g and linear expansion dL from 1.60 to 4.83 mm.

When the GGBS addition is used, the analysis of pore size distribution indicates a lower capillary pore content and a general reduction in the average pore size. As a result, high pressures are generated that damage the concrete structure. The specimens are frozen in water and the continuous contact with water allows the supply of additional water from the outside and an increase in the degree of saturation. This leads to the ice lens formation and, as a result of cyclic freezing-thawing, to the phenomenon of micro-ice-lens pumping. Passing the critical degree of saturation, even locally, is the major factor in microcrack formation, which, as freeze-thaw cycles continue, leads to the opening of the initially tight structure, taking up an additional amount of water, and as a result, to the failure of the concrete structure. In the early stage of deterioration, the mass of concrete first increases then decreases due to mass losses.

**Figure 8:**
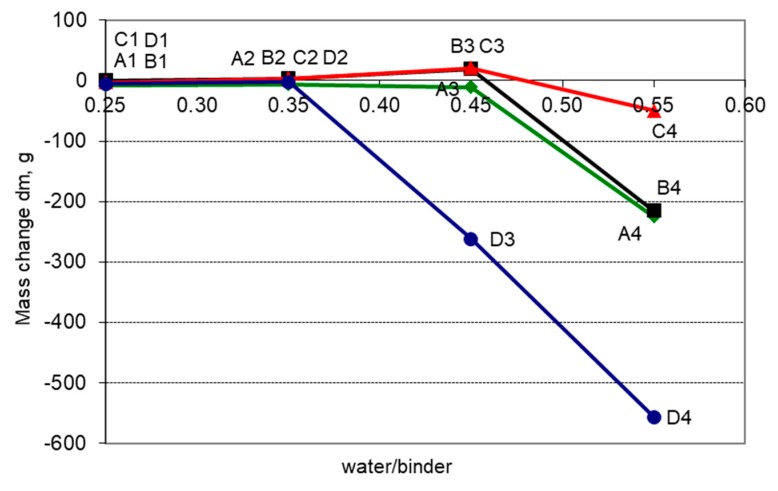
Influence of the w/b ratio and the GGBS content on mass loss, dm.

The change in mass dm after 300 freeze-thaw cycles depending on the w/b ratio and the slag content is shown in Figure 8. In the case of concretes with the slag content lower than 30%, there is a clear tendency indicating that the mass losses of the specimens increase when the w/b ratio is greater than 0.45. Much larger mass losses occur with the content of GGBS greater than 50%.

**Figure 9:**
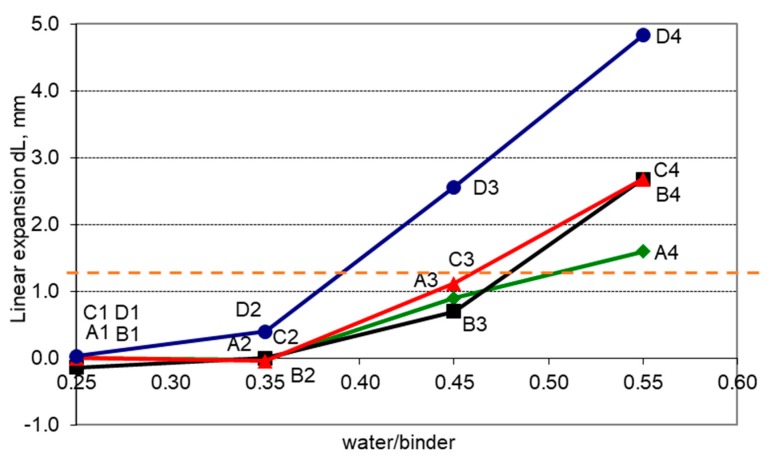
Influence of w/b ratio and GGBS content on specimen linear expansion dL.

The influence of the w/b and slag content in the binder on the development of internal damage in non-air-entrained concrete was also analyzed (Figure 9). The specimen linear expansion dL after 300 freeze-thaw cycles was assumed to be the basis for inferring about the extent of concrete damage. 

The smaller the w/b ratio, the more resistant the concrete is to internal deterioration. Low GGBS content of 20–30% in the binder does not affect the freeze-thaw resistance. It can be seen that an increase in the amount of GGBS to 40–60% clearly reduces frost resistance. With a given w/b ratio, the increase in the slag content causes greater damage to the concrete. 

Assuming the linear expansion dL_cr_ = 1.3 mm as the criterion of freeze-thaw resistance of concrete, the critical ratio (w/b)_cr_ for concrete made with cement containing varying slag contents was determined based on Figure 9. The graph shows that as the GGBS content increases, the value of the w/b ratio in the binder allowing it to meet the dL_cr_ criterion decreases. Whereas for CEM I Portland cement, the critical w/b value will be about 0.49, for the GGBS cement with a slag content greater than 50%, the (w/b)_cr_ will be about 0.38.

**Figure 10:**
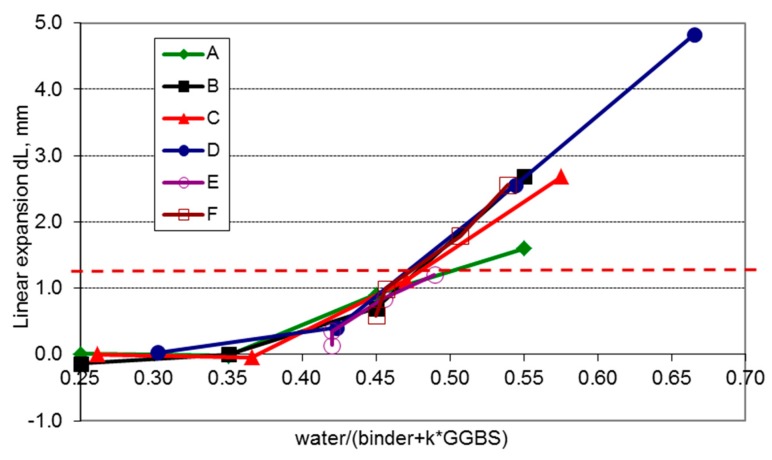
Curves of GGBS concrete deformation fitted to the reference curve (series A, CEM I).

The results obtained allowed for the estimation of the k-values for the GGBS contents in the binder (Figure 10). Once more, the deformation of the reference concretes (series A) made with CEM I cement (reference curve) was used as the basis for the analysis. The analysis was performed for the specimens with ready-made GGBS cement (series B, C, and D) and for the specimens made with the binder containing CEM I cement and GGBS addition (series E and F). The ready-made GGBS cements were regarded as CEM I to which GGBS was added. By changing the k-values through trial and error, the plots of the deformation curves for the tested concretes were adjusted so that they “coincided” with the reference curve. Analysis shows that the GGBS content in the binder affects the k-value, which decreases with increasing GGBS content (Table 3). This confirms the observations of Sanjuan and others. With a GGBS content in the binder of 40% or below, the k factor assumes the value of 0.6 or above, whereas for GGBS content exceeding 40%, the k-value is 0.5–0.6.

From the presented tests of resistance to internal cracking of non-air-entrained XF3 concretes made with binders containing different slag contents, it appears that high-GGBS addition to concrete reduces its freeze-thaw resistance performance through the linear expansion of concrete specimens. With regard to concrete internal damage, adopting w/b = 0.45 seems safe, provided that the GGBS content is 40% or below.

## 5. Conclusions

With regard to frost-resistant concrete design, one of the variants adopted in the EN 206 standard is the concept of the coefficient (k-value), which takes into account the amount of additive in the design composition of concrete by adjusting the w/c ratio. It is common belief that the coefficient (k-value) is determined for type II additive with Portland cement, CEM I, not with ready-to-use cements, such as CEM III/A and CEM III/B. However, a decrease in frost-resistance (internal cracking) is observed when both ready-made cements containing GGBS and more than 40% slag additions are used. The results discussed in this paper confirm the above statement. The fixed value of k = 0.6 is another area of controversy.

The paper proposes an approach to determining the k-value. Several series of concretes made with a binder of different GGBS contents were subjected to 300 freeze-thaw cycles and strain curves dL = f (w/b) were determined for each series. By adjusting the curve of the GGBS-containing concretes to the reference curve for Portland cement concretes, the k-values were determined for particular amounts of slag in the binder. The analysis indicates that the k-value decreases with an increase of the GGBS content in the binder. With a slag content of 40% or below in the binder, the k-value is from 0.6 to 1. When the amount of slag in the binder increases over 40%, the k-value is 0.5–0.6. The authors of this study can confirm that the proposed approach to determining the k-value within the context of concrete frost durability is a simple and effective approach.

It is important to note that the conclusions formulated here pertain to the materials used in the present study. Further research, which will be necessary to verify the relationships obtained, should include slag-containing cements of different strength classes and those derived from different sources.

## Figures and Tables

**Table 1:** Basic physico-chemical properties of the cement and GGBS used in the tests.

Property	CEM I 42, 5RHSR NA	CEM II/A-S 42, 5N	CEM II/B-S 32, 5R	CEM III/A 32, 5N	CEM I 42, 5RNA	Ground Granulated Blast Furnace Slag (GGBS)
Concrete series	A	B	C	D	E, F	E, F
GGBS, %	0	13	28	53	0	-
Density g_c_, g/cm^3^	3.14	3.04	3.02	2.96	3.15	2.88
Blaine’s surface area, cm^2^/g	3421	3547	3700	4113	3200	4445
SiO_2_, %	21.9	20.4	23.9	27.7	20.58	40.1
Al_2_O_3_, %	3.7	5.4	5.3	6.0	5.18	7.0
Fe_2_O_3_, %	4.8	2.7	2.5	1.7	2.56	0.3
CaO, %	63.2	60.6	58.6	52.5	63.7	44.8
MgO, %	1.0	2.2	2.6	4.8	0.82	5.9
SO_3_, %	2.5	2.9	2.8	2.7	2.8	-
Compressive strength Rc-2, MPa	18.2	27.4	23.6	-	22.1	-
Compressive strength Rc-28, MPa	44.2	56.1	55.9	45.2	54.1	-

**Table 2:** Experimental design.

Binder	GGBS, % Binder Mass	Water/Binder				
		0.25	0.35	0.45	0.55	0.42
CEM I	0	A1	A2	A3	A4	-
CEM II/A-S	13	B1	B2	B3	B4	-
CEM II/B-S	28	C1	C2	C3	C4	-
CEM III	53	D1	D2	D3	D4	-
CEM I	0	-	-	-	-	E1
CEM I+GGBS	15	-	-	-	-	E2
CEM I+GGBS	35	-	-	-	-	E3
CEM I+GGBS	55	-	-	-	-	E4
CEM I	0	-	-	F1	-	-
CEM I+GGBS	20	-	-	F2	-	-
CEM I+GGBS	40	-	-	F3	-	-
CEM I+GGBS	60	-	-	F4	-	-

**Table 3:** Experimentally determined k-values.

**GGBS, %**	0	15	20	30	40	50–60
**k**	0	1	0.9	0.8	0.6	0.5–0.6
